# Associations of Class-Time Sitting, Stepping and Sit-to-Stand Transitions with Cognitive Functions and Brain Activity in Children

**DOI:** 10.3390/ijerph16091482

**Published:** 2019-04-26

**Authors:** Emiliano Mazzoli, Wei-Peng Teo, Jo Salmon, Caterina Pesce, Jason He, Tal Dotan Ben-Soussan, Lisa M. Barnett

**Affiliations:** 1 School of Health and Social Development, Faculty of Health, Deakin University, Geelong VIC 3220, Australia; 2 Physical Education and Sports Science (PESS) Academic Group, National Institute of Education (NIE), Nanyang Technological University, Singapore 637616, Singapore; weipeng.teo@nie.edu.sg; 3 School of Exercise and Nutrition Sciences, Faculty of Health, Deakin University, Geelong VIC 3220, Australia; jo.salmon@deakin.edu.au; 4 Institute for Physical Activity and Nutrition (IPAN), Deakin University, Geelong VIC 3220, Australia; 5 Department of Movement, Human and Health Sciences, Italian University of Sport and Movement, 00135 Rome, Italy; caterina.pesce@uniroma4.it; 6 Cognitive Neuroscience Unit, School of Psychology, Deakin University, Geelong VIC 3220, Australia; jason.he@deakin.edu.au; 7 Cognitive Neurophysiology Laboratory, Research Institute for Neuroscience, Education and Didactics, Patrizio Paoletti Foundation, 06081 Assisi, Italy; research@fondazionepatriziopaoletti.org

**Keywords:** sedentary behaviour, executive functions, attention, brain activity, children, school-based, class time

## Abstract

Previous research showed that children’s physical activity is positively related to executive functions, whilst screen time shows negative associations. However, it is unclear how school-based sitting time and transitions from sitting to standing relate to cognition. We investigated the relationship between class time sitting/stepping/sit-to-stand transitions and cognitive functions in Grade 1–2 children. Overall, 149 children (7.7 ± 0.6 years old, 54% boys) participated. Measures included class time sitting/stepping/sit-to-stand transitions and: (i) response inhibition (i.e., response time and accuracy); (ii) lapses of attention; (iii) working memory; and (iv) brain activity (cortical haemodynamic response). Linear mixed-models, adjusting for age, sex, and clustering at the classroom level, found that more sitting time was associated with higher lapses of attention (*β* = 0.12, *p* < 0.05). Children who stepped more had quicker inhibition response time (*β* = −0.95, *p* < 0.01); however, they were less accurate in their responses (*β* = −0.30, *p* < 0.05) and this was also observed with sit-to-stand transitions (*β* = −0.26, *p* < 0.05). No associations were found with brain activity. In conclusion, reducing and breaking up sitting may help keep children focused, but the evidence regarding response inhibition is unclear.

## 1. Introduction

International guidelines recommend that children engage in physical activity for at least 60 min every day [1]. Spending no more than 2 h a day in recreational screen time and reducing and regularly breaking up prolonged sitting time are also advised [2]. These recommendations are based on the health-enhancing benefits of being physically active [3]. Physical activity benefits the physical, cognitive, social, psychological and academic aspects of children’s lives [4,5,6]. There is also growing evidence of the detrimental health effects of sedentary behaviour [7]. Sedentary behaviour—defined as energy expenditure ≤1.5 metabolic equivalent while being awake in a sitting or reclining posture [8]—includes behaviours such as recreational screen time (watching television or playing videogames), time spent sitting in a car, reading, playing board games, or doing homework, etc. 

A strong body of evidence in adults links sedentary behaviour to cardiovascular disease, diabetes, obesity and cognitive decline, independent of physical activity levels [9,10,11,12,13]. As such, those who are sedentary for long uninterrupted periods, even if they meet physical activity guidelines are at risk of poorer health outcomes. Emerging evidence suggests that children with higher screen time have poorer cardio-metabolic health outcomes [7,14,15]. Evidence of associations between objectively assessed sedentary behaviour and health in young people is less consistent. For example, a systematic review of associations between objectively assessed sedentary time with health outcomes among children and adolescents found little evidence of associations [16]. Most of the 29 studies in this review were observational (cross-sectional or longitudinal), only one study used a direct measure of sitting, and just three studies had assessed associations between sedentary time and cognitive outcomes.

Furthermore, long bouts of sitting may be detrimental for children’s academic performance. Yet, only one study has investigated this relationship in children; with objectively measured sedentary time negatively associated with reading and arithmetic skills in boys aged between 6 and 8 years [17]. Conversely, the same study [17] found that sedentary time was positively associated with arithmetic skills in Grade 2 girls. Furthermore, other studies that objectively measured weekly [18,19] or non-school leisure sedentary time [20] in older children (9–13 years old) failed to find significant associations with academic achievement. It may be that the task engaged in while sitting is important for different cognitive outcomes or it might be that there is only a weak or no association between sitting time and cognitive outcomes.

In spite of the evidence which suggests breaking up sitting is important, children are largely sedentary and do not meet physical activity guidelines [16,21]. The school setting is where children spend most of their waking hours [22], with children in Australia and the UK spending approximately 70% of class time being sedentary [23]. It is possible to reduce and break up children’s prolonged sitting using simple classroom-based strategies: active breaks, active lessons, and/or re-designed environments (i.e., standing desks) [24]. Even though these strategies are feasible [25], low-cost [26,27], and generally effective in reducing sedentary behaviour [28,29] and may enhance children’s cognitive functions [30], schools still favour traditional approaches to teaching. 

Cognitive functions refer to a number of mental processes related to perception, attention, memory, motor control, thinking and language [31]. Executive functions—or executive control—is an umbrella term that refers to the processes that require concentration, where intuition alone would unlikely produce desirable outcomes [32]. Miyake and colleagues [33] have identified three core executive functions: inhibition, working memory and cognitive flexibility. According to neurodevelopmental researchers [32], the combined use of these core functions allows us to reason, solve problems and make decisions—skills considered critical in life [34]. At the neurophysiological level, executive functions have been associated with neural activity of the prefrontal neocortex [35].

The wide body of evidence supporting the positive relationship between physical activity and executive functions in children has led scientific experts in this field to publish a position statement on this issue [4]. At the same time, a recent systematic review raised the issue of inconsistent cognitive outcomes of physical activity interventions for children [36]. Although, Alvarez-Bueno et al. [37] reviewed 36 physical activity interventions and compared the effects of physical activity on cognition by grouping them by cognitive domain (i.e., non-executive cognitive functions, core executive functions and metacognitive functions) concluding that physical activity interventions, particularly programs aimed at increasing physical activity time, are likely to facilitate children’s cognitive development.

Executive functions are commonly measured using behavioural tests, where participants perform a task that largely relies on the use of a specific executive function (e.g., working memory). For example, the Go/No-Go task [38] is a cognitive test designed to measure a person’s ability to inhibit impulsive responses; the performance at the task (i.e., response time and accuracy rate) is used to measure the person’s response inhibition ability. Additionally, exceptionally long response times at a cognitive task can be interpreted as lapses of attention. These lapses of attention, or momentary distractions, can affect goal-oriented behaviour [39]. Whilst this temporary inability to maintain attention can be useful in certain situations (e.g., when identifying a threat [40]) it is generally seen as problematic in the educational context [41].

The prefrontal cortex is the brain region associated with executive functions [42]. Thanks to recent advancement in cognitive neuroscience, it is now possible to use a number of neuroimaging techniques (e.g., functional near-infrared spectroscopy (fNIRS) [43]) to objectively investigate event-related brain activity. In brief, fNIRS measures cortical haemodynamic changes within a specific cortical area [44]. Some of the advantages of using fNIRS—instead of other available neuroimaging techniques, such as functional magnetic resonance imaging (fMRI)—include the low-cost, the portability and the relative resistance to motion artefacts. This makes this one of the preferred methods for assessing brain activity in children and for conducting research in an ecologically valid environment [43]. A few studies have used fNIRS to assess the relationship between physical activity and brain activity in children [45]. For example, Lambrick et al. [46] assessed children’s brain activity on the dominant side of the prefrontal cortex and children’s performance at a cognitive task designed to assess inhibition control (Stroop task [47]) before and after a 15-min continuous or intermittent moderate-intensity running exercise. After the exercise session, children’s performance at the cognitive task improved and oxygen availability in the prefrontal cortex (i.e., oxy-haemoglobin and total haemoglobin) increased, explaining almost 50% of the variability of the cognitive performance. A cross-sectional study [48] compared prefrontal cortex activity in children with low weekly moderate-to-vigorous physical activity levels vs. those with high moderate-to-vigorous physical activity levels, finding no differences between groups. 

To our knowledge, no studies have explored the effects of sitting on objective measures of brain activity and/or lapses of attention in children. Furthermore, only four studies have investigated the effects of objectively measured sedentary time, with accelerometers, and cognitive functions in children [49,50,51,52], finding mixed associations. A cross-sectional study found that children’s sedentary time was related to worse inhibition control [52]. Syvaoja and colleagues [50] found that longer objectively measured sedentary time was associated with better attention, but they did not find associations in the other cognitive domains. Aadland et al. [49] found that higher objectively measured sedentary time was associated with better executive functions. Finally, a longitudinal study [51], revealed that longer objectively measured sedentary time was associated with better scores using a composite cognitive measure of verbal reasoning. None of these four studies assessed sedentary time with inclinometers, which directly measure sitting. 

In sum, the literature reflects a lack of clarity in terms of the relationship between objectively assessed sedentary time and different aspects of cognitive function. No studies have previously explored the association between objectively measured class time sitting (i.e., time spent sitting in the classroom during school hours, excluding recess and lunch), using inclinometers, and cognitive functions in any population. Therefore, the aim of this exploratory study was to investigate how class time sitting, stepping, and sit-to-stand transitions relate to response inhibition, working memory, lapses of attention and brain activity in Grade 1–2 children—aged between 6 and 8 years. We hypothesised that longer sitting during class time will be associated with less efficient executive functions and frontal brain activity, and longer lapses of attention; opposite associations were hypothesised for stepping during class time and sit-to-stand transitions.

## 2. Materials and Methods

This cross-sectional study was conducted using the baseline data of the study “Active breaks in the classroom to improve thinking skills”, collected in October 2017. This trial was registered in the Australian New Zealand Clinical Trials Registry (registration number ACTRN12618002034213). The trial was retrospectively registered as the researchers did not complete this prospectively, but thought it would still be useful.

### 2.1. Ethics Approval

All subjects gave their informed consent for inclusion before they participated in the study. The study was conducted in accordance with the Declaration of Helsinki, and the protocol was approved by Deakin University Human Research Ethics Committee on the 25th of January 2017 (2016-382) and the Department of Education and Training of Victoria (2016_003257).

### 2.2. Participants

**Figure 1:**
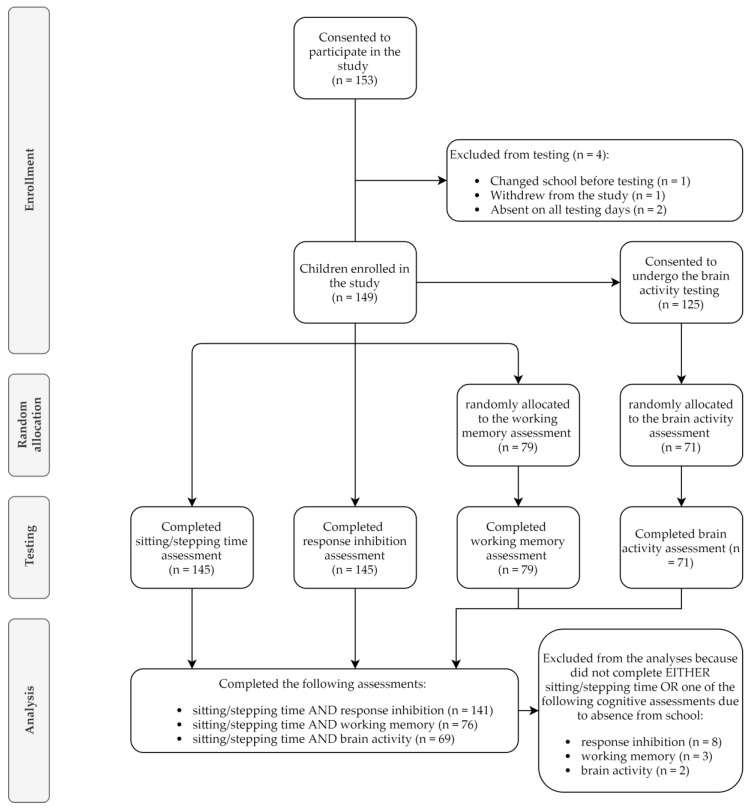
Recruitment according to the Consolidated Standards of Reporting Trials (CONSORT) flow diagram [55].

A convenience sample of three primary schools within the metropolitan area of Melbourne, Australia, were recruited in 2017. Parents or guardians of Grade 1–2 children were provided with a plain language study description and consent form, invited to complete a demographic survey and provide consent for their child to participate in the study. Overall, there was parental consent for 153 children from 15 classrooms. One child changed school before study commencement, one withdrew and nine were absent on at least one of the assessment days. A total of 149 children completed the sitting/stepping time or the response inhibition assessments; 141 completed both. To be included in the study children had to be between 6 and 8 years old. No other exclusion criteria were used. All consenting children could undergo the assessments, although brain activity was only measured in children who provided the required additional consent. Overall, 15 children were reported to have medical conditions or developmental issues: asthma (*n* = 3), sensory processing disorder (*n* = 2), dyslexia (*n* = 2), attention-deficit/hyperactivity disorder (*n* = 2), autism spectrum disorder (*n* = 2), speech delay (*n* = 1), hypercalciuria (*n* = 1) and global developmental delay (*n* = 1); one condition was not specified by parents/guardians. Since these children did not alter the pattern of results, they were included to enhance the generalisability of the study outcomes. Due to the time requirements of conducting working memory and brain activity assessments, these were limited to a random sub-sample of 79 and 71 children respectively—of which 76 and 69 had also a measure of sitting/stepping time (see Figure 1 for recruitment flow diagram).

### 2.3. Procedures

Consenting children wore an activity monitor (i.e., *activ*PAL^TM^) for two school days to measure their sitting/stepping time. Two researchers fitted the monitors at the start of the school day and instructed children to wear them until the end of the day. This was purposely done during days that did not include physical education or school sport to capture a typical school day across the different classrooms, avoiding confounding effects of those activities. The monitors were collected by the researchers at the end of each school day. Data were downloaded at the end of the second day of assessment in each school. Children’s cognitive functions (i.e., response inhibition and working memory) and brain activity were measured by two to three researchers in a quiet room within the school premises, within the same week but on school days not involving their sitting/stepping time assessments.

### 2.4. Measures 

#### 2.4.1. Demographics

Children’s demographic characteristics—i.e., date of birth, language spoken at home, parents’ country of origin, education, occupation and income—were collected via a parent survey.

#### 2.4.2. Sitting Time, Stepping Time, and Sit-to-Step Transitions

Children’s sitting and stepping time was measured with *activ*Pal™ (PAL Technologies Ltd., Glasgow, UK), a small and light-weight battery-operated uniaxial inclinometer (35 × 53 × 7 mm; 15 g), normally worn on the thigh, which collects data on the limb position sampling at 10 Hz. The device was enclosed in a customised pocket and fitted at the mid-point on the child’s front thigh using an adjustable elastic band. *Activ*PAL™ monitors have demonstrated good validity for measuring time spent sitting or stepping, and sit-to-stand transitions in school-aged children [53], even in a school classroom setting [54].

#### 2.4.3. Response Inhibition

**Figure 2:**
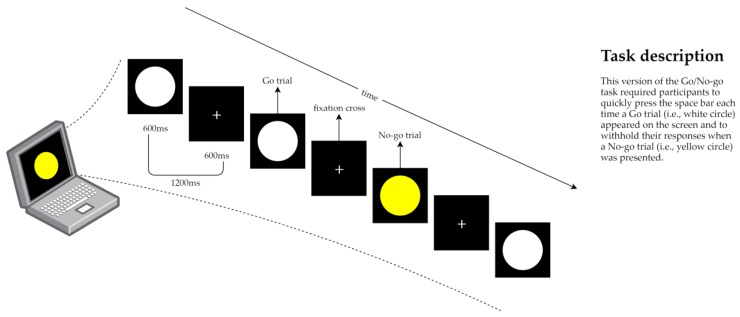
Graphical representation of the Go/No-Go task protocol, based on the paradigm originally proposed by Donders [38].

Response inhibition was assessed using the well-validated Go/No-Go task [56], originally proposed by Donders [38], and programmed in E-Prime 2.0 Software (Psychology Software Tools, Pittsburgh, PA, USA). The task, displayed on a 14.1-inch Lenovo laptop (Lenovo Group Ltd., Beijing, China), presented participants with a pseudo-random series of visual stimuli—white or yellow circles on a black background. Participants were told that when presented with a white circle (Go-trial), they were to respond as quickly and as accurately as possible by depressing the space bar on the keyboard. Alternatively, when presented with a yellow circle (No-go trial), they should have tried to withhold their responses. Each stimulus was preceded by a fixation cross and both the fixation cross and the visual stimulus were presented for 600 ms (see Figure 2). All participants completed a practice block which included 20 trials—70% Go-trials and 30% No-go trials. After the practice block, participants completed a test blocks containing 100 trials—again, 70% Go-trials and 30% No-go trials. Both practice and test blocks began and ended with Go-trial fillers—five and 15, respectively—designed to allow the participant to settle into the task [57]. The responses to the filler trials were not considered for subsequent analyses.

#### 2.4.4. Lapses of Attention

Temporary lapses of attention are momentary distractions, which are known to interfere with goal-directed behaviour. The most common way of measuring lapses of attention involves fitting an ex-Gaussian distribution—a right-skewed-looking bell curve resulting in the sum between a Gaussian distribution and its exponential—over each participant’s reaction time data [58]. Response times under the exponential side of the distribution constitute the metric of lapses of attention, commonly referred as τ (tau). Research shows that people exhibiting frequent lapses of attention show difficulties in performing everyday tasks and this may negatively affect a person’s well-being [59]. A strong body of evidence highlights that children with attention-deficit/hyperactivity disorder (ADHD) show larger and more frequent lapses of attention [60].

#### 2.4.5. Working Memory

Working memory was measured using the iPad-based National Institutes of Health (NIH) Toolbox List Sorting Working Memory Test [61] validated for children and adolescents (7–17 years). The test consists of two blocks; each block included two practice trials designed to help children become more familiar with the test. The first test block asks children to recall the names of food or animals visually and orally presented, and to list them back to the researcher in size order from the smallest to the largest. In the second block children were asked to perform the same task but listing all the food first and then the animals. The minimum number of items presented in each trial is two and the maximum is seven. An item is added to the list following each correct response. In case of an incorrect response, a new list with the same number of items is presented. Two consecutive errors mark the end of the block/test. The test results’ raw score ranges from 0 to 26. The software also calculates an uncorrected standardised score—comparing the participant’s result to an American nationally representative normative sample—and an age corrected score—calculated by adjusting the uncorrected standardised score by age. The uncorrected standardised score was used for the analyses.

#### 2.4.6. Cortical Haemodynamic Response (Brain Activity)

Dorsolateral prefrontal cortex haemodynamic response was measured using a wearable and wireless battery-operated single-channel functional near-infrared spectroscopy (fNIRS; probe size: 58 × 28 × 6 mm) (PortaLite, Artinis Medical Systems, The Netherlands). While near-infrared light can safely penetrate human tissues (e.g., skin and bones), it is absorbed or scattered by pigmented compounds (chromophores) [44]. The system takes advantage of these properties to calculate changes in the oxygenated and deoxygenated haemoglobin (O_2_Hb and HHb, respectively). The PortaLite device constantly emits light at 760 and 850 nm wavelengths between a transmitter and three aligned receivers, placed at 30, 35 and 40 mm from the transmitter. The measure of light attenuation is used to calculate the changes in O_2_Hb and HHb occurring in the brain region corresponding to where the probe is placed, relatively to an arbitrary value set as zero, using a modified Beer–Lambert Law [62]. The device remotely transmits the acquired data to a nearby computer where the information is recorded using the Artinis proprietary software (Oxysoft, Artinis Medical Systems, Elst, The Netherlands) and marked in real time by a researcher, to distinguish the occurring events (i.e., test/rest periods). Participants were fitted with the PortaLite probe on their forehead in the area corresponding to their dorsolateral prefrontal cortex and were asked to perform a computer-based cognitive test, similar to the one described in Section 2.4.3. 

**Figure 3:**
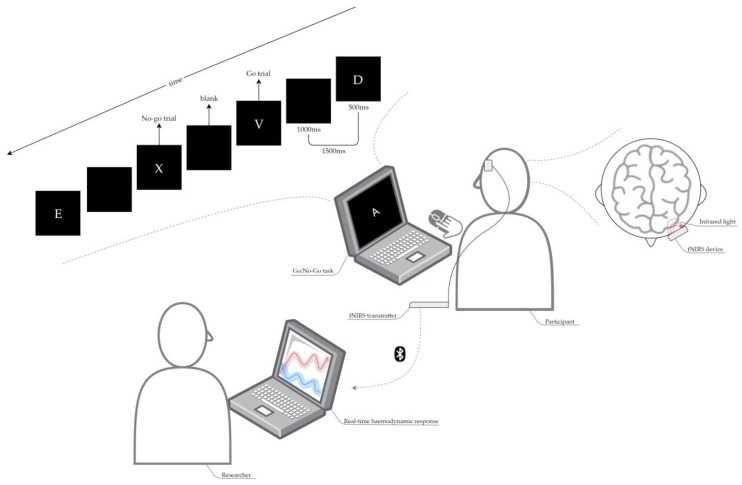
A graphical representation of the fNIRS setup. Each participant involved in the brain activity assessment completed the following protocol: (A) it was explained to the participant how to perform the cognitive task (i.e., Go/No-Go task) and completed a practice session; (B) the participant was fitted with the fNIRS device probe on the forehead in the area corresponding to their dorsolateral prefrontal cortex; (C) the participant completed the Go/No-Go task while the researcher concurrently recorded the task-related haemodynamic response.

The cognitive test is a Go/No-Go task [38] programmed with E-Prime Software and designed to match the brain activity assessment requirements and duration: due to its high inter- and intra-individual variability, haemodynamic response is usually measured multiple times for each task block type and then average values are used for statistical analysis. This version of the Go/No-Go task required children to click the left mouse key for each letter presented on the computer screen during a “Go” block. Alternatively, children were instructed to click the left mouse key for each letter except the letter ‘X’ within the “No-Go” block. After the instructions, children had a practice with both block types—including five trials each—to help them familiarise with the task. Brain activity data were not collected during this phase. The test session included five Go blocks and five No-Go blocks lasting 30 s each, presented in alternation, each including 20 trials and alternated with 30 s rests. The letter X was presented in each test block at a 1/2 ratio. Children wore the fNIRS monitor on their forehead for the total duration of the computer-based task, approximately 10 min (Figure 3).

### 2.5. Data Management

#### 2.5.1. Sitting Time, Stepping Time and Sit-to-Step Transitions

*Activ*PAL™ data were downloaded in 15 s epochs using the proprietary software (*activ*PAL™ Professional, version 7.2.32) and then processed using a previously designed MS^®^ Excel macro (Microsoft Corporation, Redmond, WA, USA). Specific data calculated for the analyses included class (excluding recess and lunch) and school daily time spent sitting and stepping, and the frequency of transitions from sitting to standing. To be included in analyses, children had to have worn the *activ*PAL™ for at least one of the two school days. A 50% wear time criterion was applied to identify valid class time periods [63]; data consisting of ≥20 min of consecutive zeros were identified as non-wear time [64]. Each sitting/stepping/sit-to-stand variable was standardised according to the wear time to allow comparison between subjects with different wear times. This was done by dividing each variable by the wear time (265.32 ± 11.04 min) and multiplying it by the total class time (270 min), excluding recess and lunch breaks. The average daily class time sitting and stepping, and average sit-to-stand transitions across the two school days were calculated and used for the analysis.

#### 2.5.2. Response Inhibition

Response inhibition data from all subjects were merged in one file using E-Prime and then processed using MS^®^ Excel. Children’s responses to the practice block and to the Go-trial fillers and premature responses (≤150 ms) were not included in the analyses [57]. Each participant’s performance at this test was summarised as average reaction time of the correct response (Go) and the proportion of accurate inhibited responses (No-Go).

#### 2.5.3. Lapses of Attention

For each participant, lapses of attention (*τ*) was extracted using R Statistical Software (Version 3.5.1, The R Foundation for Statistical Computing, Vienna, Austria), R Studio (Version 1.1.463., RStudio Inc., Boston, MA, USA), and the package “retimes” [65]. The package “retimes” [65] is a collection of density functions, developed for R Statistical Software, which allows to fit an ex-Gaussian distribution—a “right-skewed normal distribution”—on response time data. This specific procedure and the estimation of the distribution parameters—mean (*µ*), standard deviation (*σ*), and the exponential component of the distribution (*τ*)—were performed using the function “mexgauss”, which implements the methods of moments as previously described by Heathcote et al. [58].

#### 2.5.4. Brain Activity

Brain activity data were initially processed with the proprietary software to ensure the correctness of all event markers inputted on the day of data collection. Then, all the data were imported in MATLAB (MATLAB R2017a, MathWorks, Natick, MA, USA) and processed using the hemodynamic optically measured evoked response (HomER2) user interface, previously developed by Huppert and colleagues [66]. Artefacts were removed using the techniques described by Brigadoi and colleagues [67]. This process involved the following steps: raw data were first converted to optical density changes; an algorithm of filters was applied to the data to detect motion artefacts; detected artefacts were corrected using a principal component analysis (PCA) filter; bandpass filters (high pass filter = 0.010 Hz; low pass filter = 0.20 Hz) were applied to the data to clear it from low and high frequencies noise; optical density data were converted to concentration changes. For each participant, the average changes in O_2_Hb and HHb occurring during Go and No-Go blocks were extracted for data analysis. The average values were extracted between 10 and 30 s of each test block, to accommodate for the haemodynamic response time course [68]. A more detailed data processing description, including the utilised pipeline values, and the MATLAB script are attached in Appendix A (Table A1). The cognitive data collected during the brain activity assessment with the Go/No-Go task was processed following the method already described in Section 2.5.2.

#### 2.5.5. Demographic Data Processing 

Demographic data were entered in separate data sheets and did not require additional processing. All the data sets were imported into STATA 15.0 (Stata Statistical Software, Release 15, StataCorp LLC, College Station, TX, USA) and merged into one file for data analysis.

### 2.6. Data Analysis

Summary statistics of the sample demographic information, sitting/stepping time, cognitive performance, and brain activity were calculated. Continuous data were assessed for normality with histograms, Q–Q plots and by examining skewness and kurtosis values. Pairwise Pearson’s correlation coefficients between independent variables (sitting/stepping time/sit-to-stand transitions) and outcome variables (cognitive functions/brain activity) were calculated to explore the initial unadjusted relationships between variables. Overall, 18 linear mixed-model analysis, adjusted for age and sex, and nested at the classroom level, were performed between each independent variable: (1) sitting, (2) stepping or (3) sit-to-stand; and each cognitive/brain activity outcome: (1) response inhibition reaction time; (2) response inhibition accuracy; (3) lapses of attention; (4) working memory; (5) O_2_HB; and (6) HHB. The models including brain activity data as an outcome were also adjusted for the performance level on the cognitive task completed during the assessment. STATA 15.0 was used for the analyses. Given the exploratory nature of the study and to better inform future research all results are presented without performing a *p*-value correction (e.g., Bonferroni correction).

## 3. Results

The age of children included in the analyses was 7.7 ± 0.6 years (54% boys). Most participants (91%) spoke English at home. Descriptive statistics of the parental education, occupation and income are presented in Table 1.

The summary statistics of children’s class time sitting, stepping and sit-to-stand transitions, response inhibition, lapses of attention, working memory and brain activity are presented in Table 2. 

**Figure A1:**
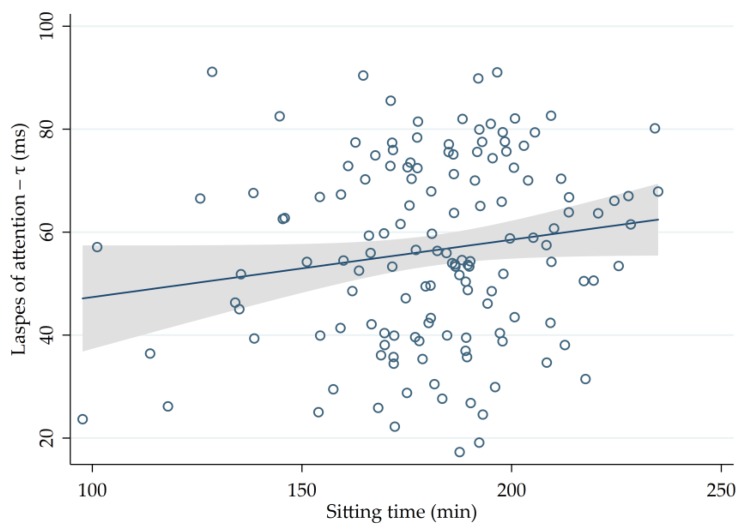
Scatterplot showing the correlation between sitting time and lapses of attention (*r* = 0.16, *p* = 0.06).

**Figure A2:**
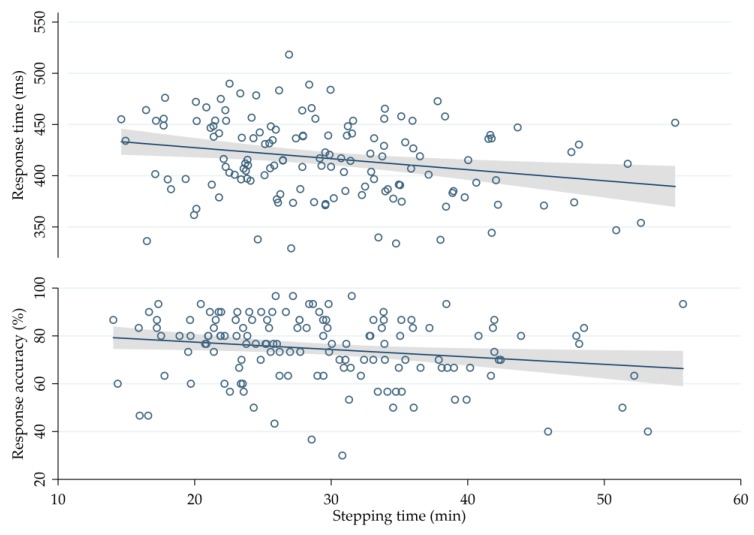
Scatterplot showing the significant correlations between class time stepping and response inhibition reaction time (*r* = −0.24, *p* < 0.005) and accuracy (*r* = −0.19, *p* < 0.05).

The correlation between sitting time and lapses of attention approached significance (*r* = 0.16, *p* = 0.06—Figure A1). Statistically significant correlations, although weak, were found between stepping time and response time at the Go-trials (*r* = −0.24, *p* < 0.01) and accuracy at the No-Go trials (*r* = −0.19, *p* < 0.05) (Figure A2); and between sit-to-stand transitions and response inhibition accuracy (*r* = −0.17, *p* < 0.05). Pearson’s correlations coefficients between sitting, stepping, sit-to-stand, cognitive performance and brain activity data are presented in Appendix A Table A2. 

The results from the mixed models showed that sitting time was significantly positively associated with lapses of attention (*β* = 0.12, 95% CI (0.01, 0.23), *p* < 0.05; intraclass correlation coefficient (ICC) = 0.005). For each additional minute children spent sitting in the classroom there were 0.12 ms more lapses of attention on average. Stepping was negatively associated with inhibition response time (*β* = −0.95, 95% CI (−1.66, −0.26), *p* < 0.01; ICC < 0.001), and negatively associated with response inhibition accuracy (*β* = −0.30, 95% CI (−0.56, −0.04), *p* < 0.05; ICC < 0.001). Each additional minute spent stepping during class time corresponded to 0.95 ms faster correct responses at the Go/No-Go task, on average. However, higher daily stepping time and more sit-to-stand transitions also corresponded to poorer response inhibition accuracy. No significant associations were found between daily sitting/stepping time, or sit-to-stand transitions and working memory or brain activity. The results of the mixed models are presented in Table 3.

## 4. Discussion

To our knowledge, this is the first study to assess the relationships between class time sitting, stepping and sit-to-stand transitions, and cognitive functions and brain activity. Overall, the outcomes of our study suggest that some aspects of daily sitting time, stepping and sit-to-stand transitions during class time are related to some cognitive functions in Grade 1–2 children. Daily class time spent sitting was associated with lapses of attention, and stepping was associated with faster reaction time, but also with poorer accuracy on the response inhibition test. The latter was also observed in relation to daily sit-to-stand transitions. 

Haapala and colleagues [17] found negative associations between objectively measured sedentary time and measures of reading and writing skills in boys aged between 6 and 8 years. Considering the similar age of our sample and the evidence suggesting a causal link between executive functions and academic achievement [69], we hypothesised that a negative association between sitting and executive functions could also be found. Our finding regarding lapses of attention aligns with Haapala and colleagues’ finding, confirms our hypothesis, and suggests that children between 6 and 8 years old may need to break up prolonged sitting more often than they currently do. In a study exploring the perceived determinants of sedentary behaviour, Hidding et al. [70] interviewed a group of children and parents in the Netherlands and found that children considered sitting as the “norm” and the most appropriate behaviour at work and play. In Australia, class time is typically uninterrupted for about two consecutive hours between the start of the day and recess and a further two hours between recess and lunch. Simple and inexpensive classroom-based activities, such as active breaks, can help children and teachers preventing that most of the morning is spent sitting [30,71]. Although teachers consider classroom sitting as a way to keep children focused and attentive [72], our study results suggest that this may not be the case. The implementation of strategies to reduce and break up children’s sitting during class time is low-cost. For example, Erwin et al. [26] reported that the equipment necessary to conduct active breaks costed only US$180 per school. Similarly, redesigning classroom environments with standing desks was indicated as relatively low-cost compared to traditional desks and chairs [27]. Moreover, implementing movement in the classroom can be relatively easy because it requires little or no equipment, little effort by the teachers and may also be curriculum-integrated [73]. For example, teachers could implement a simple 5-min active break as a transition from one subject to the other—for example, a sequence of simple movements and stretches (e.g., twisting, bending, knee lifts, squatting and jumping on the spot) that children can imitate standing behind or beside their desks. An example of a curriculum-integrated activity is the game “Alphabetising” [74]: children start standing on a line and the teacher asks them to position themselves in alphabetical order by first name without talking and keeping at least one foot on the line at all times. Although they are effective, low-cost and easy to implement, the delivery of these strategies in the school environment requires more evidence of positive cognitive and academic outcomes, the creation of appropriate environmental conditions, and a change in school leaders’ and teachers’ beliefs of the incompatibility between physical activity, learning outcomes and academic achievement [75].

Three previous studies [49,50,51] found mixed results between sedentary time and some measures of cognitive functions—e.g., working memory and verbal fluency. Overall, two of the studies [49,50] assessed different executive functions using a number of cognitive tests, each reflecting a specific function (e.g., Digit Span test to assess working memory [76]), whereas one study [51] used a measure which seemed to reflect the overall cognitive-verbal abilities (i.e., British Ability Scale) rather than executive functions. None of the measures used in previous studies examining the relationship between sedentary time and cognition were designed to assess response inhibition.

In contrast with our findings, Syvaoja et al. [50] found that prolonged sedentary time was associated with better sustained attention, van der Niet et al. [52] found that greater sedentary time was associated with worse inhibition control (interference), and Aadland and colleagues [49] found significant positive associations between sedentary time and inhibition control in boys, and working memory in girls. By contrast, and consistent with our study, Syvaoja et al. [50] found no association between sedentary time and working memory. Despite these apparent inconsistencies in the findings, various differences in the way cognitive functions were measured should be highlighted. Syvaoja et al. [50] measured attention with the intra-extra dimensional set shift within the Cambridge Neuropsychological Test Automated Battery (CANTAB), a composite measure of cognitive flexibility and attention based on the Wisconsin Card Sorting test. By contrast, our measure of lapses of attention is more directly related to the momentary losses of attention. With respect to working memory, it is important to note that Baddeley [77] identified three components: a central executive and two storage systems, the phonological loop and the visuospatial sketchpad. Aadland et al. [49] measured working memory with the Wechsler intelligence scale for children (WISC-IV), Backward Digit Span. This measure is supposed to assess the phonological loop with auditory inputs, although because of the low mental manipulation demand, some researchers use it as a measure of short-term memory rather than working memory [78]. Syvaoja et al. [50] measured working memory with a Corsi task part of CANTAB, a measure of visuospatial working memory. The working memory test used in our study is designed to assess the phonological loop by providing combined visual and auditory inputs. The measure-specific differences highlighted above, might explain the inconsistent findings on the same cognitive construct (i.e., working memory). 

Similarly, the cognitive task used by Aadland and colleagues [49] and van der Niet et al. [52] to measure inhibition control (i.e., Stroop task) was originally designed to measure selective attention and cognitive flexibility [79], and is also supposed to assess a particular cognitive aspect of inhibition known as interference control [47]. Moreover, the children included in those studies [49,50,51,52] were older compared to our study sample and some of the findings refer to cognitive outcomes, such as verbal fluency or sustained attention, that we did not assess. Importantly, the measures of cognition used in the previous studies investigating the relationship between sedentary and cognition in children [49,50,51,52] were not specifically designed to capture children’s ability to refrain responses (i.e., response inhibition) and/or brain activity, which make comparisons difficult to draw. Furthermore, Aadland and colleagues [49] argue that objectively measured sedentary time might be a poor predictor of cognitive functions because it does not provide information about the specific behaviour that the child is performing while sitting. However, considering that our study was conducted during school hours it could be assumed that children were engaged in scholastic activities during that time, suggesting that prolonged sedentary scholastic activities may interfere with children’s cognitive performance.

Our study also revealed that children who accumulated more stepping time during daily class time exhibited faster response time, which is consistent with previous research [37]. Conversely, given that interventions have consistently found that physical activity benefits children’s inhibition accuracy [37], the fact that in the current study class time stepping and sit-to-stand transitions was associated with poorer accuracy at the response inhibition test was unexpected. While children who spent less time sitting showed higher capability to stay focused (i.e., less lapses of attention), more time spent stepping was associated with a shift toward higher inhibitory speed. The shift in inhibition might represent a prerequisite for remaining on-task while quickly suppressing undesired thoughts and impulsive actions, although the cost of this is a loss of inhibition accuracy. Furthermore, this supports the notion of independent effects of sedentary behaviour and physical activity, extending it from the physical health domain to the cognitive domain. Novel research on these nuances should be conducted to clarify how sedentary behaviour and physical activity are unitedly or independently associated with cognitive functions.

Another possible reason for the unexpected finding regarding inhibition accuracy could be the mismatch of data collection timing. Although sitting/stepping time/sit-to-stand transitions and cognition/brain activity were assessed during the same week, the data were not collected on the same day to avoid excessive disruption to the schools. The mean daily sitting, stepping and sit-to-stand data were collected on days which did not include physical education or school sport. However, the same criterion was not applied when measuring cognitive functions. It could have been that children had a physical education lesson or school sport on the day that cognitive performance or brain activity was assessed. This may have had small but positive acute effects on cognitive functions [80]. Similarly, children’s cognitive performance may have been influenced by the preceding classroom activities. 

Our study did not show any significant associations between daily class time sitting, stepping and sit-to-stand transitions, and brain activity. While this did not confirm our hypothesis, considering that brain activity was measured using a single-channel fNIRS device it is possible that we overlooked significant cortical activations in other near brain regions. This is; therefore, another limitation of our study. The only other cross-sectional fNIRS study in school children [48] that examined the relationship between moderate-to-vigorous physical activity and brain oxygenation in the left and right prefrontal cortex also failed to show an association. Given the convenience sample and the fact that most children were coming from high-income families, the findings from this study may not apply to the whole population of Grade 1–2 children. An additional study limitation is the lack of inclusion of measures of sleep, weight status and food intake, which have been associated with cognitive and academic performance in children and adolescents [81,82,83].

All previous studies investigating how objectively measured sedentary behaviour relates to cognition [9,49,50,51,52] assessed sedentary time using accelerometers (i.e., ActiGraph; LLC, Pensacola, FL, USA), with a cut-off value of 100 counts per minute to identify sedentary time. Although this is generally considered acceptable [84], compared to inclinometers (e.g., *activ*PAL™), accelerometers cannot detect postural changes and may classify both sitting and standing time as sedentary time [84]. Therefore, the employment of inclinometers to objectively assess sitting/stepping time and sit-to-stand transitions is a strength of the study. Additional strengths include the large sample size for a study investigating brain activity in children and the use of a measure of brain activity in an ecologically valid setting.

There is paucity of studies exploring how different types of physical activity or sports may differently influence children’s cognitive functioning [85]. Future studies investigating the effects of various types of physical activity and/or sedentary behaviour on cognitive functions are needed; these should consider using a more representative sample, testing cognitively-engaging forms of physical activity and sports, expanding the measure of sitting time to more than two school days, aligning the days of assessment of the sitting/stepping/sit-to-stand transition data with the cognitive functions and brain activity data, and using an intervention design to allow causal associations to be explored.

## 5. Conclusions

This study aimed to identify whether sitting/stepping time and/or sit-to-stand transitions were associated with cognitive functions in schoolchildren. Children who spent more daily class time sitting showed more lapses of attention—i.e., they appeared more easily distracted. On the other hand, greater stepping time was associated with quicker inhibition response time and lower accuracy. Sitting in the classroom for long periods should be avoided, because it hinders children’s ability to stay focused. Replacing sitting with stepping may enhance children’s response inhibition speed while improving their attention, which might ultimately increase their response inhibition accuracy. However, since evidence around the inhibitory measures of cognition was mixed, further research should clarify this aspect. In light of our findings regarding lapses of attention and considering the growing evidence that suggests positive effects of physical activity on children’s cognitive functions [37] and negative associations between sedentary behaviour and children’s physical health [7], classroom-based strategies to reduce and break up class time sitting can be recommended. Although, whether these strategies do indeed positively impact on cognition, needs to be further tested empirically over a sustained period of time. 

## Figures and Tables

**Table 1:** Participants’ parental country of origin, education, occupation and income (count (percentages)).

Socio-Economic Characteristics	Father/Guardian *n* (%)	Mother/Guardian *n* (%)
*n*	145	149
**Country of Origin**		
Australia	95 (65.5)	103 (72.0)
Asia	23 (15.9)	28 (19.6)
Europe	18 (12.4)	7 (4.9)
Other ^a^	9 (6.2)	5 (3.5)
**Education**		
Primary school	-	1 (0.7)
Some high school	1 (0.7)	3 (2.0)
Completed high school	11 (7.7)	11 (7.5)
Technical/trade certificate/apprenticeship	16 (11.2)	8 (5.4)
University or tertiary education	114 (79.7)	124 (84.4)
Not applicable	1 (0.7)	-
**Employment status ^b^**		
Employed full time	119 (82.6)	38 (25.2)
Employed part time	13 (9.0)	72 (47.7)
Unemployed or unpaid	5 (3.5)	7 (4.6)
Home-duties full time	1 (0.7)	25 (16.6)
Student	-	6 (4.0)
Not applicable	2 (1.4)	1 (0.7)
Other ^c^	4 (2.8)	2 (1.3)
**Combined income**		
<AUD 30,000	4 (2.9)	
AUD 30,000–59,000	12 (8.8)	
AUD 60,000–119,000	18 (13.2)	
AUD 120,000–180,000	50 (36.8)	
>AUD 180,000	52 (38.2)	

^a^ Canada, New Zealand, South Africa, Sudan, Turkey, Uganda, USA; ^b^ due to multiple responses the total number of values reported for these characteristics may exceed the total number of respondents; ^c^ passed away, self-employed, casual. *n*, number. Total percentage values may not equal 100 due to rounding. Missing responses are not reported.

**Table 2:** Summary statistics (mean ± standard deviation) of daily class time sitting/stepping and sit-to-stand transitions, response inhibition, lapses of attention, working memory and brain activity.

Children’s Data	*n*	Mean ± SD
Daily class time sitting (min)	145	181.89 ± 25.29
Daily class time stepping (min)	145	29.37 ± 8.60
Daily class time sit-to-stand (freq.)	145	39.94 ± 8.94
Response inhibition		
Response time (ms)	145	417.10 ± 38.01
Accuracy (percentage)	145	74.32 ± 13.90
Lapses of attention—*τ* (ms)	145	56.83 ± 18.09
Working memory (standardised test score)	79	88.39 ± 11.96
Brain activity		
O_2_Hb ^a^ (µmol/L)	71	1.60 × 10^−7^ ± 6.25 × 10^−7^
HHb ^b^ (µmol/L)	71	−6.01 × 10^−8^ ± 2.68 × 10^−7^

^a^ oxy-haemoglobin (O_2_Hb); ^b^ deoxy-haemoglobin (HHb). *n*, number. Freq., frequency.

**Table 3:** Mixed model results regarding associations between daily sitting, stepping and sit-to-stand transitions and the cognitive and brain activity outcome variables—adjusted for sex and age.

Outcome Variables	Independent Variables		
	Daily Sitting Time (Min)	Daily Stepping Time (Min)	Daily Sit-to-Stand Transitions (Freq.)
**Cognitive functions**			
Inhibition accuracy			
*β* 95% CI	0.03 (−0.57, 0.12)	−0.30 * (−0.56, −0.04)	−0.26 * (−0.52, −0.01)
Residuals ICC ICC 95% CI	7.03 × 10^−15^ (7.03 × 10^−15^, 7.03 × 10^−15^)	1.46 × 10^−21^ (1.46 × 10^−21^, 1.46 × 10^−21^)	2.76 × 10^−15^ (2.76 × 10^−15^, 2.76 × 10^−15^)
Inhibition response time			
*β* 95% CI	0.03 (−0.21, 0.28)	−0.95 ** (−1.66, −0.26)	−0.41 (−1.12, 0.30)
Residuals ICC ICC 95% CI	1.41 × 10^−16^ (1.41 × 10^−16^, 1.41 × 10^−16^)	7.81 × 10^−26^ (7.81 × 10^−26^, 7.81 × 10^−26^)	4.45 × 10^−20^ (4.45 × 10^−20^, 4.45 × 10^−20^)
Lapses of attention (*τ*)			
*β* 95% CI	0.12 * (0.01, 0.23)	−0.17 (−0.51, 0.18)	−0.18 (−0.51, 0.15)
Residuals ICC ICC 95% CI	0.005 (3.30 × 10^−10^, 0.999)	0.028 (0.001, 0.489)	0.036 (0.002, 0.382)
Working memory			
*β* 95% CI	0.01 (−0.09, 0.11)	−0.06 (−0.35, 0.23)	−0.08 (−0.41, 0.24)
Residuals ICC ICC 95% CI	1.68 × 10^−20^ (1.68 × 10^−20^, 1.68 × 10^−20^)	1.41 × 10^−19^ (1.41 × 10^−19^, 1.41 × 10^−19^)	2.63 × 10^−20^ (2.63 × 10^−20^, 2.63 × 10^−20^)
**Brain activity**			
O_2_Hb ^a^			
*β* 95% CI	1.38 × 10^−9^ (−3.80 × 10^−9^, 6.56 × 10^−9^)	3.24 × 10^−9^ (−1.29 × 10^−8^, 1.93 × 10^−8^)	−9.36 × 10^−9^ (−2.50 × 10^−8^, 6.24 × 10^−9^)
Residuals ICC ICC 95% CI	3.81 × 10^−24^ (3.81 × 10^−24^, 3.81 × 10^−24^)	3.97 × 10^−16^ (3.97 × 10^−16^, 3.97 × 10^−16^)	2.79 × 10^−15^ (2.79 × 10^−15^, 2.79 × 10^−15^)
HHb ^b^			
*β* 95% CI	−1.03 × 10^−9^ (−3.28 × 10^−9^, 1.23 × 10^−9^)	2.89 × 10^−9^ (−4.78 × 10^−9^, 1.06 × 10^−8^)	6.40 × 10^−9^ (−5.98 × 10^−11^, 1.29 × 10^−8^)
Residuals ICC ICC 95% CI	0.158 (0.025, 0.580)	0.154 (0.024, 0.576)	0.140 (0.019, 0.578)

^a^ oxy-haemoglobin (O_2_HB); ^b^ deoxy-haemoglobin (HHB); * *p* < 0.05, ** *p* < 0.01. ICC, intraclass correlation coefficient; CI, confidence interval.

## Appendix A

**Table A1:** Brain activity data processing pipeline values and description.

Process	Specific	Value	Description
HMR intensity to OD	n/a	n/a	The intensity data (number of time points multiplied by the number of data channels) are divided by the mean, and then converted to the change in OD.
HMR motion artefact remover by channel	Time motion window (s) Time mask (s) SD threshold Amplitude threshold	0.5 1.0 50.0 0.10	These filters identify and remove motion artefacts. Segments of data channels that exhibit a change that are greater than: (i) the SD threshold multiplied by the SD of the intensity data, and/or (ii) the indicated amplitude threshold, within an indicated time range, are marked as artefacts for +/− the mask time.
HMR motion correction PCA filter	Number of principal components	0.97	This function uses PCA to filter the segments identified as motion artefacts, according to the number of principal components to remove from the data.
HMR band pass filter	High pass filter Low pass filter	0.010 0.20	This perform bandpass filters: high pass filter frequency (Hz), typical values between 0 and 0.02; low pass filter frequency (Hz), typical values between 0.5 and 3.
HMR OD to concentrations	Partial pathlength factors	6.0 6.0	For each wavelength, partial pathlength factors are identified. Typical values are around 6.
HMR block average	Time range (s)	−5.0 30.0	This part of the process calculates the block average for each condition within the defined time range.

HMR, haemo-dynamic response; OD, optical density; SD, standard deviation; PCA, principal component analysis.

MATLAB processing script:

- dod = hmrIntensity2OD(d);
- [tIncAuto,tIncChAuto] = hmrMotionArtifactByChannel(dod,t,SD,tIncMan,0.5,1,50,0.1);
- [dod,svsMotion,nSVMotion] = hmrMotionCorrectPCA(SD,dod,tIncAuto,0.97);
- dod = hmrBandpassFilt(dod,t,0.01,0.2);
- dc = hmrOD2Conc(dod,SD,[6 6]);
- [dcAvg,dcAvgStd,tHRF,nTrials,dcSum2] = hmrBlockAvg(dc,s,t,[−5 30]);

**Table A2:** Pairwise Pearson’s correlation coefficients between sitting, standing and stepping data, and cognitive functions and brain activity data.

Cognitive Functions/Brain Activity	Sitting Time	Stepping Time	Sit-to-Stand Transitions
Inhibition RT	0.03	**−0.24**	−0.06
Inhibition ACC	0.07	**−0.19**	**−0.17**
Lapses of attention (*τ*)	**0.16**	−0.10	−0.08
Working memory	0.03	−0.05	−0.09
fNIRS—O_2_Hb	−0.02	0.11	−0.01
fNIRS—HHb	−0.03	0.01	0.11

RT, reaction time; ACC, percentage of accurate responses; WM, working memory; O_2_Hb, oxy-haemoglobin; HHb, deoxy-haemoglobin; fNIRS, functional near-infrared spectroscopy. Correlation coefficients with *p* < 0.1 are marked in bold.
